# Evaluation of alternative antimicrobial strategies in high-performance pigs for managing endemic *Mycoplasma hyopneumoniae* populations

**DOI:** 10.1007/s11250-026-05028-3

**Published:** 2026-04-07

**Authors:** Ricardo Yuiti Nagae, David Emilio S. N. Barcellos, Karine Ludwig Takeuti, Ana Paula Gonçalves Mellagi, Tais Regina Michaelsen Cé, Neimar Cavazinni, Cintia Kunrath, Anelcir Scher, Jonatas Wolf, Fernando Pandolfo Bortolozzo, Rafael da Rosa Ulguim

**Affiliations:** 1 Setor de Suínos, Faculdade de Veterinária - Universidade Federal do Rio Grande do Sul, Porto Alegre, Rio Grande do Sul 91540-000 Brazil; 2 Seara Alimentos Ltda, Itajaí, Santa Catarina Brazil; 3 Faculdade de Medicina Veterinária - Universidade Feevale, Campo Bom, Rio Grande do Sul Brazil

**Keywords:** Enzootic pneumonia, Lung lesion, Antibiotic free, Nursery phase, Finishing phase

## Abstract

This study evaluated pig production performance in herds with *M. hyopneumoniae* by replacing in-feed antimicrobials with the herbal extract carvacrol during nursery and finishing phases. A total of 324 piglets were allocated across three treatments: ATM-Free (without antimicrobial); HE (carvacrol); and ATM (antimicrobial). They were housed in the nursery from 21 to 63 days of age (doa), then transferred to finishing and slaughtered at 175 doa. Piglets were weighed and feed consumption was measured at 21, 28, 35, 42, 49, 56, and 63 doa in the nursery and at 75, 98, 107, 128, 135, 152, 161, and 174 doa in the finishing phase. Lung macroscopic lesions were evaluated at slaughter. Body weight (BW), average daily weight gain (ADG), and mortality/removal rate at nursery end did not differ between the HE and ATM treatments. However, feed-to-gain (F/G) was slightly worse in HE compared to ATM at nursery. The HE and ATM-Free treatments did not differ in ADG and BW, but HE was better than ATM-Free in F/G at nursery. Lung lesions score tended to be higher in ATM-Free compared to the ATM treatment. However, the frequency of lungs with a low lesion score did not differ among treatments. Removing antimicrobials from feed for pig populations endemic to M. hyopneumoniae reduces production performance during nursery and finishing phases and tends to cause larger affected lung areas suggestive of pneumonia at slaughter. Carvacrol can be an alternative to antimicrobials without relevant impacts on performance, except for the removal rate in the finishing phase.

## Introduction

*Mycoplasma hyopneumoniae* causes enzootic pneumonia (EP), a chronic disease that reduces weight gain, and increase feed conversion ratio promoting economic losses in the pig industry (Maes et al. 2018; Pieters and Maes 2019). Control strategies include vaccination, prophylactic antimicrobial use (ATM), acclimation, and production flow segregation from lactation to finishing (Garza-Moreno et al. 2018; Maes et al. 2018). Even though vaccination is the most common control strategy (Garza-Moreno et al. 2018; Maes et al. 2018) reducing clinical symptoms and severity of lung lesions (Baccaro et al. 2006; Sibila et al. 2007), it does not prevent infection (Maes et al. 2008; Pieters et al. 2010). Therefore, the use of ATM treatments is frequently required for EP control (Garza-Moreno et al. 2018).

The effectiveness of ATM, combined with vaccination and biosecurity programs, in reducing damage caused by M. hyopneumoniae has been demonstrated by previous studies (Stipkovits et al. 2003; Pallarés et al. 2015; Stingelin et al. 2022). However, the use of ATM should be limited and only justified for treating pigs showing clinical signs of EP to avoid the development of ATM resistance (Garza-Moreno et al. 2018; Maes et al. 2020). Essential oils (EO) have been used as an alternative to ATM in animal production (Kim et al. 2008; Yang et al. 2015; Baschieri et al. 2017) to improve immune status and pig performance (Choo et al. 2006; Li et al. 2012; Tan et al. 2015; Zou et al. 2016). One example of an EO is carvacrol, which has been reported to act as an antimicrobial, anti-inflammatory, and antioxidant compound to enhance intestinal health and improve growth performance (Jamroz et al. 2006; Costa et al. 2013; Cheng et al. 2018; Nostro and Papalia 2012; Suntres et al. 2015). Moreover, the therapeutic effects of carvacrol are related to the control of respiratory disorders, as demonstrated in five studies in humans and 12 in rodents (Carvalho et al. 2020). Most of the previously cited studies have focused on mechanistic outcomes, short-term responses, or evaluations within a single production phase. Limited information is available under commercial field conditions, particularly when assessing performance across production phases, from nursery through the growing–finishing phase until slaughter. Therefore, the current study contributes by evaluating productive performance through to slaughter, thereby supporting its practical applicability in commercial swine systems.

The evaluation of carvacrol as a prophylactic ATM treatment in swine herds with endemic *M. hyopneumoniae* has not been effectively explored. Thus, the objective of our study was to assess the performance of pigs given feed supplemented with the herbal extract carvacrol as a prophylactic in-feed alternative to ATM to control *M. hyopneumoniae* under endemic conditions during the nursery and finishing phases in commercial pig farms.

## Materials and methods

All management and procedures used in this experiment were approved by the Ethics Committee on Animal Use of the Universidade Federal do Rio Grande do Sul under protocol number 38,906.

### Animals, housing, and handling

The study was performed in a nursery and a finishing farm located in Rio Grande do Sul, Brazil. Piglets were weaned from a commercial sow farm endemic to *M. hyopneumoniae*, located in Santa Catarina, Brazil, with an inventory of 2,300 sows (Agroceres PIC). The farm was free from Porcine Reproductive and Respiratory Syndrome (PRRS), Transmissible Gastroenteritis (TGE), and Porcine Epidemic Diarrhea (PED).

The piglets were weaned at 21 (± 1) doa, transferred to a nursery farm, and housed in pens with 12 piglets/pen until 63 doa. The nursery barn housed 1,800 piglets in pens with a partially slatted plastic floor, manual feeder, and one nipple drinker. The temperature and air quality were controlled by a wood furnace and a double curtain lining. The same piglets were moved to a finishing farm at 63 doa and housed nine pigs/pen until slaughter (175 doa). The finishing farm housed 360 pigs and consisted of a partially slatted concrete floor, manual feeders, one nipple drinker/pen, and a double curtain lining to control the temperature and air quality. Nurseries and finishing farms used all-in/all-out protocol and a downtime of seven days prior to housing.

All piglets were vaccinated at weaning for Porcine circovirus 2 (Ingelvac^®^ CircoFLEX, Boehringer Ingelheim, Brazil), *M. hyopneumoniae* (Hyogen, CEVA, Brazil) and *Lawsonia intracellularis* (Porcilis^®^ Ileitis, MSD, Brazil) according to the manufacturers’ guidelines. The nutritional protocol followed the farm’s standard for each productive phase, with feed formulation and nutritional levels according to the NRC (2012). Regardless of the treatment received in the study, animals exhibiting any clinical condition that required ATM use were medicated by intramuscular injection using a disposable needle, according to the manufacturer’s instructions. The ATM used to treat pigs were amoxicillin 15 mg/kg (Vetrimoxin Advance, CEVA, Brazil), enfofloxacin 7.5 mg/kg (Kinetomax^®^, Elanco, Brazil), tulathromycin 2.5 mg/kg (Draxxin^®^, Zoetis, Brazil), and ceftiofur 5 mg/kg (CEF-50, União Química Farmacêutica Nacional, Brazil).

### Experimental design

The trial was performed during the nursery and finishing phases. Three treatments were compared: (1) ATM – the use of therapeutic dosages of ATM in the feed; (2) ATM-Free – no use of ATM in the feed or water; (3) HE – the exclusive use of an herbal extract in the feed. The ATM and HE treatments were administered directly at the feed mill. Carvacrol was the component of the herbal extract administered in feed 2.0 mg/kg (Activo^®^, GRASP, Brazil) in the nursery phase and 0.65 mg/kg (Vigora Max, GRASP, Brazil) in the finishing phase. The ATM administered in the feed were tiamulin 10.0 mg/kg (Denagard™ 80%, Elanco^®^), florfenicol 2.5 mg/kg (Amphenor^®^ 50 and Sanphar^®^, Brazil), and tilmicosin 14.0 mg/kg (Hi-Bac^®^ 50, Farmabase Saúde Animal, Brazil) as described in Table 1.

**Table 1:** Description of the in-feed treatments by pig age in the nursery and finishing phases of pig production

Phase	Age (days)	Treatment		
		ATM	ATM-Free	HE
Nursery	21–28	tiamulin	-	carvacrol
	29–35	tiamulin	-	carvacrol
	36–42	-	-	carvacrol
	42–63	florfenicol	-	carvacrol
Finishing	64–75	florfenicol	-	carvacrol
	75–98	-	-	-
	98–107	tiamulin	-	carvacrol
	108–128	-	-	carvacrol
	129–135	florfenicol	-	carvacrol
	136–152	-	-	carvacrol
	153–161	tilmicosin	-	carvacrol
	162–174	-	-	carvacrol

Treatments: ATM = therapeutic antimicrobial (200 ppm tiamulin, 100 ppm florfenicol, 400 ppm tilmicosin); ATM-Free = no antimicrobial; HE = herbal extract carvacrol (48 ppm at nursery and 15 ppm at finishing phase)

A total of 600 female piglets were randomly selected at birth, tattooed, and ear tagged. At weaning, 324 piglets 20–22 doa were randomly distributed in 27 pens (nine pens/treatment) in the nursery phase according to age and weight. Each pen held 12 piglets (0.33 m^2^/pig). Each pen had partially slatted plastic floors, manual feeders, and two nipple drinkers. The temperature and air quality were controlled by a wood furnace system and double curtain lining. Feed consumption and body weight were evaluated by pen at 21, 28, 35, 42, 49, 56, and 63 doa. To maintain the same average weight per pen at the end of the nursery phase, some piglets (23, 21 and 22 piglets from the ATM, ATM-Free and HE treatments, respectively) were selected and removed from the trial. At 63 doa, the remaining piglets (*n* = 243) were moved to a finishing farm and distributed among 27 pens (nine pens/treatment). This facility had a compact floor, manual feeders, two nipple drinkers/pen, and a double curtain lining to control the temperature and air quality. In the finishing phase, each pen held nine piglets (1.00 m^2^/pig).

In the finishing phase, feed consumption and body weight were measured at 75, 98, 107, 128, 135, 152, 161, and 174 doa. Pigs were slaughtered at 175 doa. Pigs that did not gain or lose weight between weighing events were removed from the trial for the reason of low performance and counted in the removal rate. Body weight (BW), average daily weight gain (ADG), average daily feed intake (ADFI), feed-to-gain (F/G), and mortality rate were measured as performance variables, whereas lung lesions scores were measured for health status, as explained later. Additionally, during the nursery and finishing phase, the number of pigs removed due to weakness and clinical condition that required injected ATM application were recorded per treatment. The cost and the net income per pig in each treatment were estimated to perform descriptive comparisons. In this case, only the feed costs and the value of pigs at slaughter were considered.

### Sampling and lung lesion scoring

Samples were collected from 34 piglets in the ATM-Free (*n* = 17) and ATM (*n* = 17) groups. Nasal swabs (NS) were collected from piglets at 21 doa. After restraining the piglet, both nasal cavities were swabbed with a rayon swab (INLAB, Brazil) inserted into the nostrils by rotation to reach deep into the turbinates (Fablet et al. 2010). Tracheobronchial secretions (TBS) were collected at 63, 100, and 153 doa. After restraining the animal and placing a mouth gag, a catheter designed for use in post-cervical artificial insemination (Magaplus S^®^, Zaragoza, Spain) was inserted into the trachea (Takeuti et al. 2022) to collect the sample. Each sample was stored in a Falcon tube (10 mL) containing 1.0 mL of phosphate buffered saline. At slaughter, 220 lungs (ATM *n* = 76; HE *n* = 72; ATM-Free *n* = 72) were evaluated by lung lesion scores (LLS), as described by Madec and Kobisch (1982), and total affected lung area was evaluated as described by Christensen et al. (1999). Bronchial swabs (BS) were used to collect samples at slaughter from the lungs in all treatments by rotating the rayon swabs (INLAB, Brazil) in the bronchia and then moving them up and down, as described by Pieters et al. (2009). All samples were stored at -80 °C until laboratory processing.

### Laboratory analysis

Samples were processed for DNA extraction by using the magnetic particle processor procedure with the commercial MagAttract 96 Cador Pathogen kit (QIAGEN, Germany) and processed on MAGMAX (Applied Biosytems, Foster City, CA, USA) according to the manufacturer’s recommendations. Samples of NS, TBS, and BS were individually subjected to *M. hyopneumoniae* detection by PCR (Dubosson et al. 2004). In addition, NS and BS samples were individually subjected to Influenza A detection by PCR (Zhang and Harmon et al. 2014). Samples with cycle threshold (Ct) value < 40 were considered positive either for *M. hyopneumoniae* or Influenza A. Amplifications were performed in a Fast Real-time PCR System 7500 (Apllied Biosystems™, Foster City, CA, USA).

### Statistical analysis

The statistical analyses were performed using the GLIMMIX procedure in SAS (Statistical Analysis System) software version 9.4 (SAS Institute Inc., Cary, NC, USA, 2019). Pens were considered the experimental unit. The data for BW, ADG, ADFI, and F/G were analyzed by repeated measures, using treatment, doa, and their interaction as fixed effects. Overall pig performance was analyzed in the nursery and in finishing phase by using the GLIMMIX procedure for BW, ADG, ADFI, and F/G. The amount in milligrams (mg) of ATM used per kilogram (kg) of pig was calculated for each treatment by summing the mg used and dividing by the sum of BW of all pigs in the treatment at 174 doa. The initial average body weight per pen was considered as a covariate for growth performance variables. Mortality, removal rate, and percentage of animals treated with injected antimicrobial were analyzed by the NPAR1WAY procedure and Kruskal-Wallis test. Results were considered significant when *P* ≤ 0.05 and tended to differ when 0.05 < *P* ≤ 0.10.

Descriptive analysis of the proportion of samples positive for *M. hyopneumoniae* and Influenza A were performed for each time point of collection. Lung lesion score and total affected lung area were analyzed by comparisons of means. The frequency of lesion scores was analyzed using ordinal multinomial distribution, grouped in four levels 0, 1 to 4, 5 to 9, and ≥ 10 (Hillen et al. 2014). In this case, the observational unit was the lung, and the GLIMMIX procedure included the treatments as the fixed effect.

## Results

### Nursery productive performance

**Fig. 1:**
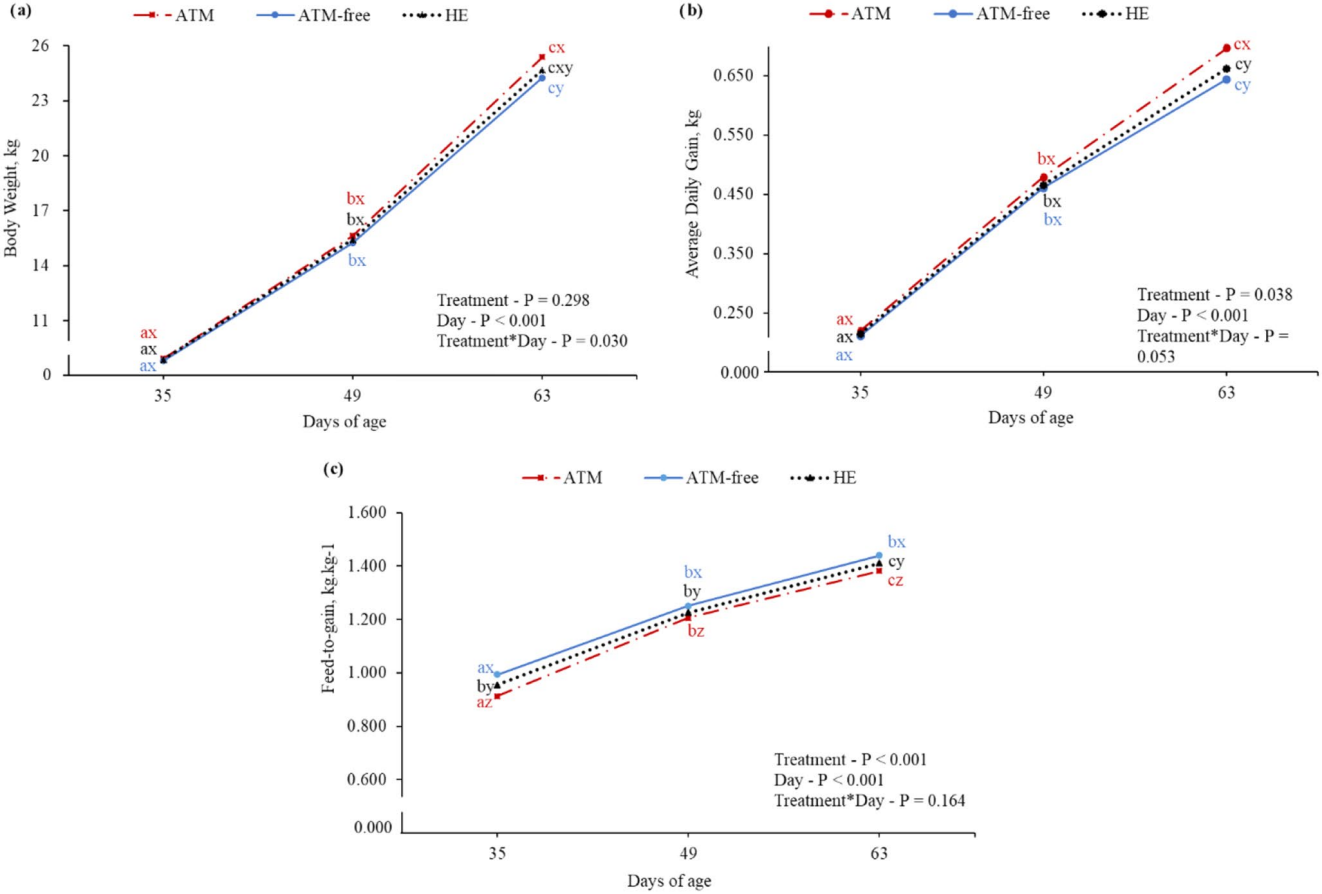
Body weight (**a**), average daily weight gain (**b**) and feed-to-gain ratio (**c**) during nursery phase in *Mycoplasma hyopneumoniae* endemic pigs per treatment. Treatments: ATM = use of antimicrobial; ATM-Free = no use of antimicrobial; HE = use of herbal extract carvacrol. Data points with different letters a–c are significantly different between sampling points (*P* ≤ 0.05). Data points with different letters x–z are significantly different among treatments (*P* ≤ 0.05)

Piglet BW increased during 35 to 49 doa in the nursery phase with no differences among treatments. However, piglets from the ATM treatment were 1.01 kg heavier (*P* = 0.030) than those in the ATM-Free (Fig. 1a) at 63 doa, and both treatments did not differ from HE treatment. A similar response was observed at 63 doa for ADG, which was higher (*P* = 0.053) in the ATM treatment compared to the ATM-Free and HE treatments, which did not differ from each other (Fig. 1b); there were no significant differences among treatments at 35 and 49 doa. There was no interaction (*P* = 0.164) between treatment and age for F/G in the nursery phase (Fig. 1c). The F/G was higher (*P* < 0.001) in the ATM treatment (1.167 ± 0.003) followed by the HE treatment (1.197 ± 0.003) and the ATM-Free treatment (1.229 ± 0.003). The F/G significantly (*P* < 0.001) increased from 35 doa (0.954 ± 0.003) to 49 (1.228 ± 0.003) and 63 doa (1.411 ± 0.003) in the nursery phase.

In the nursery phase (21–63 doa), BW (*P* = 0.031) and ADG (*P* = 0.030) were higher in the treatment ATM than in the ATM-Free treatment but not significantly different from the HE treatment. The F/G in the ATM and HE treatments was 4.2% and 2.7%, respectively, more efficient than in the ATM-Free treatment (*P* < 0.001). No piglet deaths were recorded in the nursery phase in any treatment, and removal rates did not differ (*P* = 0.777) among treatments (Table 2).

**Table 2:** Overall production performance of nursery pigs raised in herds with endemic *Mycoplasma hyopneumoniae* and treated with different in-feed antimicrobial strategies

Production variable	Treatment			SEM	*P*- value
	ATM	ATM- Free	HE		
Number of pens	9	9	9		
Body weight, kg					
21 days of age	5.97	5.93	5.99	0.451	0.996
63 days of age	25.39^a^	24.28^b^	24.64^ab^	0.278	0.031
ADG, kg	0.465^a^	0.439^b^	0.447^ab^	0.066	0.030
ADFI, kg	0.580	0.571	0.567	0.008	0.517
F/G, kg kg^− 1^	1.247^a^	1.302^c^	1.267^b^	0.004	< 0.001
Percentage of removal	4.63	3.70	5.55	-	0.777

Treatments: ATM = use of antimicrobial; ATM-Free = no use of antimicrobial; HE = use of herbal extract carvacrol. Means of average daily weight gain (ADG), average daily feed intake (ADFI), and feed-to-gain (F/G = total feed intake per pen/total weight gain per pen) across pens in the treatment are presented. SEM = standard error of the mean. Treatment parameters with different letters a–c within each production variable are significantly different (*P* < 0.05)

### Finishing productive performance

**Fig. 2:**
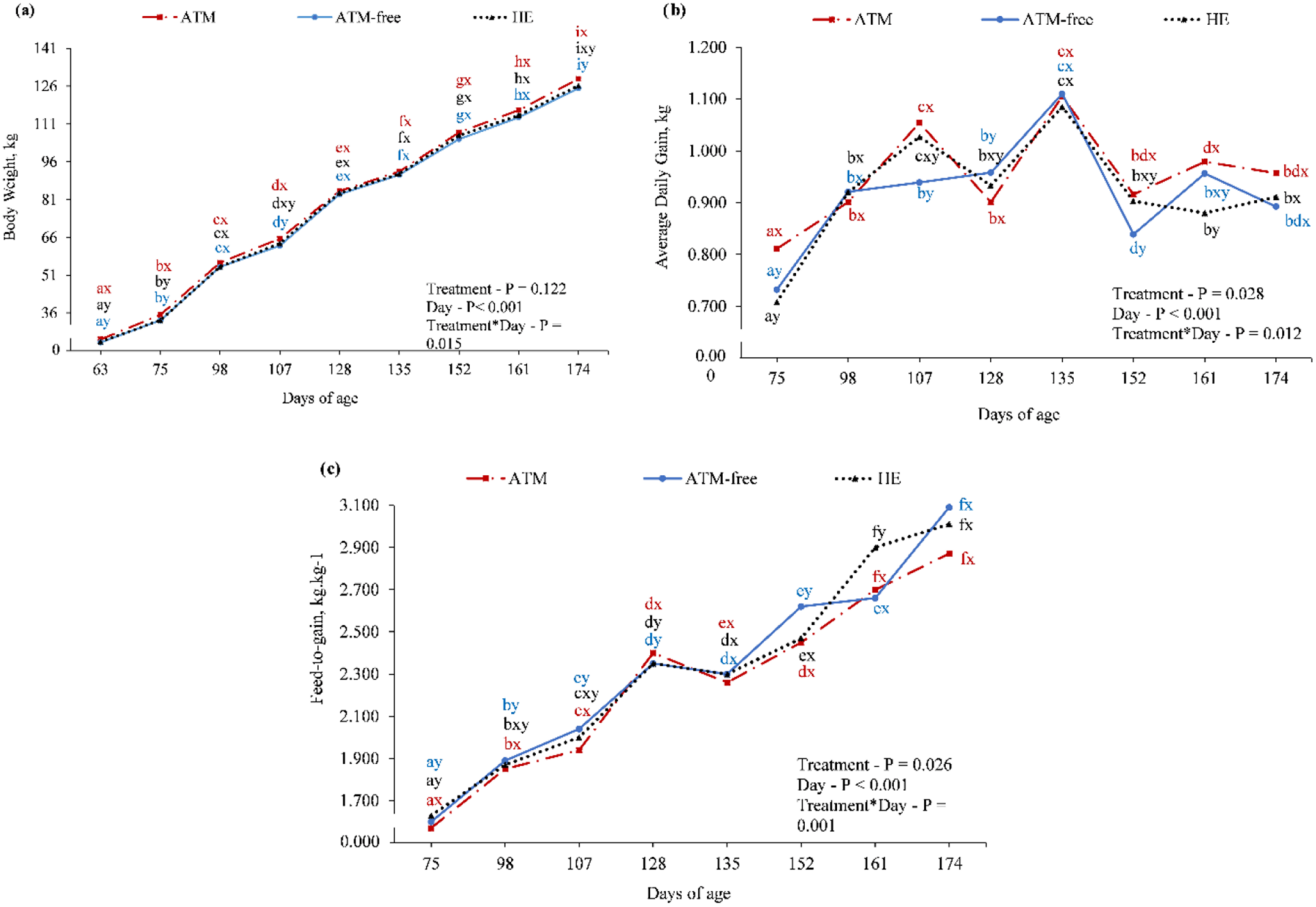
Body weight (**a**), average daily weight gain (**b**), and feed-to-gain ratio (**c**) during finishing phase in *Mycoplasma hyopneumoniae* endemic pigs per treatment. Treatments: ATM = use of antimicrobial; ATM-Free = no use of antimicrobial; HE = use of herbal extract. Data points with different letters a–i are significantly different between sampling points (*P* ≤ 0.05). Data points with different letters x–y are significantly different among treatments (*P* ≤ 0.05)

In the finishing phase, the BW was higher in the ATM treatment compared to ATM-Free and HE treatments until 75 doa. However, BW was similar from 98 to 174 doa between the ATM and HE treatments. At the end of the finishing phase (174 doa), the ATM pigs were heavier than ATM-Free pigs, but neither treatment differed from HE pigs (Fig. 2a). The ADG was variable over time in all treatments (Fig. 2b). The ATM treatment had higher ADG compared to the ATM-Free treatment at 75, 107, 128, and 152 doa. However, when compared to the HE treatment, the ATM treatment differed only at 75 and 161 doa. The use of ATM resulted in better F/G performance than ATM-Free until 128 doa, but no differences were observed at 135, 161, and 174 doa. Differences in F/G between the ATM and HE treatments were observed only at 75, 128 and 161 doa (Fig. 2c).

In the finishing phase, BW at 174 doa tended to be higher (*P* = 0.109) in the ATM treatment than in the ATM-Free treatment but similar to the HE treatment (Table 3). Although the ADG was highest (2.5%) in the ATM treatment, it did not differ significantly among treatments (Table 3). The F/G in the ATM treatment was significantly lower (*P* = 0.038) than in the ATM-Free and HE treatments, being 2.5% more efficient than ATM-Free (Table 3). The mortality rates in the ATM-Free and HE treatments were both 1.23%, whereas no piglet deaths in the ATM treatment were recorded. Cumulative removal and mortality rates in the ATM-Free and HE treatments were higher (*P* = 0.042) than in the ATM treatment (Table 3).

**Table 3:** Overall production performance of finishing pigs raised in herds with endemic *Mycoplasma hyopneumoniae* and treated with different in-feed antimicrobial strategies

Parameter	Treatment			SEM	*P*-value
	ATM	ATM- Free	HE		
Number of pens	9	9	9		
Body weight, kg					
63 days of age	25.50^a^	24.29^b^	24.59^ab^	0.267	0.011
174 days of age	128.93^a^	125.25^b^	126.19^ab^	1.222	0.109
ADG, kg	0.932	0.909	0.916	0.001	0.273
ADFI, kg	2.086	2.091	2.085	0.017	0.965
F/G, kg kg^− 1^	2.245^a^	2.302^b^	2.286^b^	0.015	0.038
Percentage mortality + removal	1.23^b^	12.35^a^	9.87^a^	-	0.042

Treatments: ATM = use of antimicrobial; ATM-Free = no use of antimicrobial; HE = use of herbal extract carvacrol. Means of average daily weight gain (ADG), average daily feed intake (ADFI) and feed-to-gain (F/G = total feed intake per pen/total weight gain per pen) across pens in the treatment are presented. SEM = standard error of the mean. Treatment parameters with different letters a–c within each production variable are significantly different (*P* < 0.05)

The net income (total value-total feed cost) of ATM-Free pigs was lower than ATM pigs by US$2.32/pig and HE pigs by US$0.88/pig (Table 4). Although the net incomes were quite similar, the ATM-Free pigs were impaired by the lower slaughter weight that negatively affected the total value of the pigs.

**Table 4:** Feed costs and mean value of pigs raised in herds with endemic *Mycoplasma hyopneumoniae* and treated during the nursery to finishing phase with diferent in-feed antimicrobial strategies

Parameter	Treatment		
	ATM	ATM- Free	HE
Feed costs			
Total feed intake, kg/pig	256.4	256.3	255.9
Feed cost, US$/pig*	97.43	97.39	97.24
Additive cost, US$/pig	1.90	0.0	0.40
Total feed cost, US$/pig	99.33	97.39	97.60
Values at industry			
Slaughter weight, kg	128.93	125.25	126.19
Total value, US$/pig**	149.55	145.29	146.38
Total value-Total feed cost, US$	50.22	47.90	48.78

Treatments: ATM = use of antimicrobial; ATM-Free = no use of antimicrobial; HE = use of herbal extract carvacrol. *Feed cost without the additives = US1.16/kg based on average value from 2015 to 2024 obtained from the Centro de Estudos Avançados em Economia Aplicada, Esalq/USP, Brazil

### Injected ATM medication

No significant differences in the use of injected therapeutic ATM were observed among treatments (*P* = 0.574), with ATM = 0.31 mg/kg, HE = 0.47 mg/kg, and ATM-Free = 0.46 mg/kg. During the nursery and finishing phases, 18.9% of all pigs received injected ATM medication (ATM-Free = 21.0%, HE = 21.0%, and ATM = 14.8%). Moreover, 85.2% of the pens in the entire trial had pigs receive injected ATM medication (ATM-Free = 88.9%, HE = 100.0%, and ATM = 66.7%).

### Mycoplasma hyopneumoniae and influenza A detection

*Mycoplasma hyopneumoniae* was not detected by PCR until 63 doa (end of the nursery phase). However, the prevalence of positive pigs increased over time, reaching 29.4% in the ATM-Free treatment and 23.5% in the ATM treatment at 100 doa, and 100% in the ATM-Free treatment and 88.2% in the ATM treatment at 153 doa. Influenza A virus was detected at 21 doa in 82.4% of the ATM-Free pigs and in 76.5% of the ATM pigs, with no additional positive cases over the remaining time. No Influenza A virus was detected in the BS collections performed at slaughter. However, *M. hyopneumoniae* was detected across all treatments: 97.2% in the ATM-Free treatment; 98.6% in the HE treatment; 97.4% in the ATM treatment.

### Lung lesion score (LLS)

**Fig. 3:**
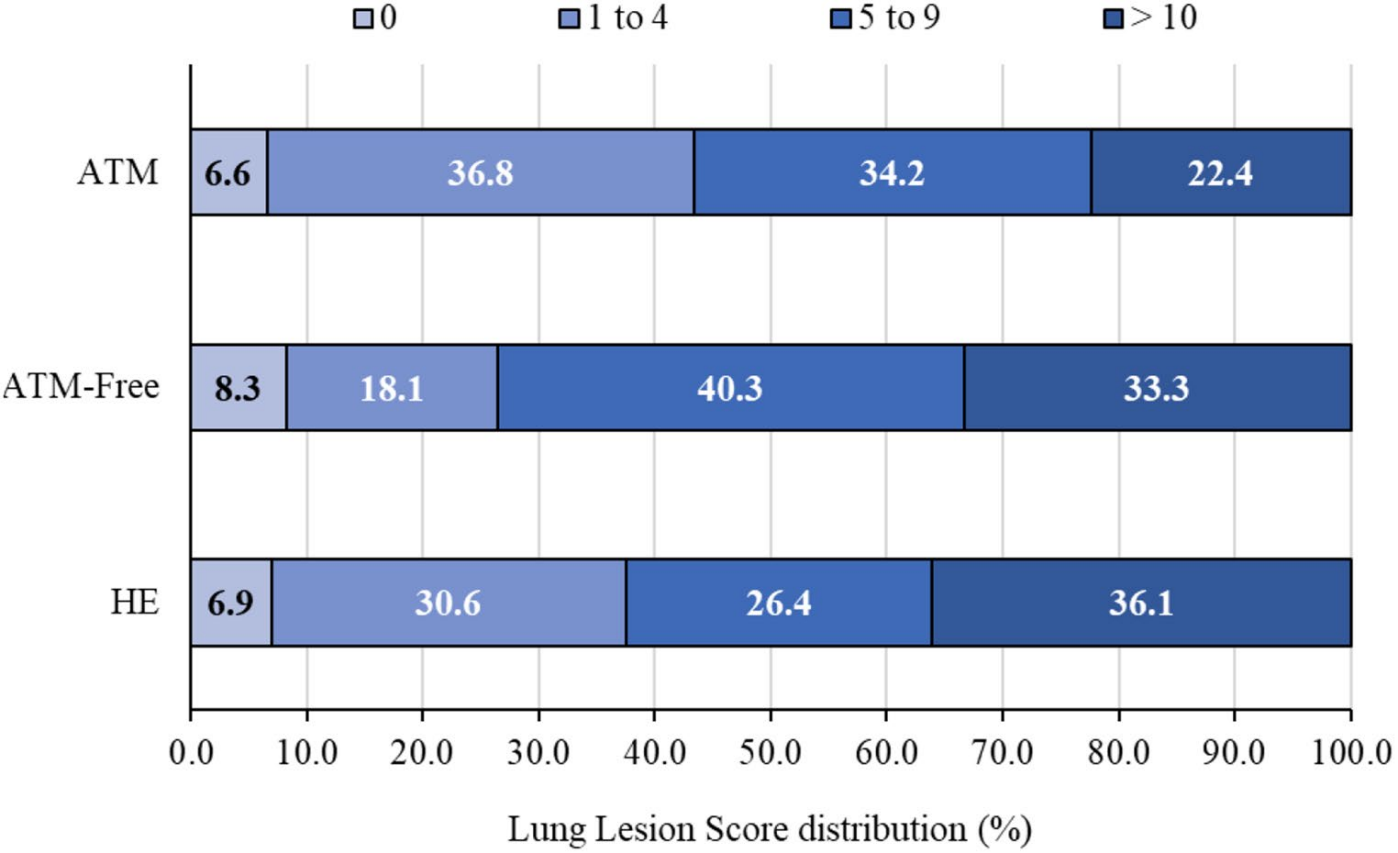
Distribution of lung lesion scores at slaughter for pigs endemic on *Mycoplasma hyopneumoniae* per treatment. Treatments: ATM = use of antimicrobial; ATM-Free = no use of antimicrobial; HE = use of herbal extract. Lung Lesion Score is represented by 0 as “no injuries” and > 10 as “highest lesion score”. No significant differences (*P* = 0.159) were observed among treatment strategies

Of the 220 lungs evaluated at slaughter, 92.7% had macroscopic lesions suggestive of EP. The average total affected lung area was 25.76% for all treatments (ATM-Free = 29.61%, HE = 26.44%, and ATM = 21.47%). The affected lung area tended to be higher (*P* = 0.096) in the ATM-Free treatment compared to the ATM treatment but not different in the HE treatment. The LLS in the ATM-Free treatment (8.29) tended to be higher (*P* = 0.095) than in the ATM treatment (6.01), while the LLS in the HE treatment (7.40) was statistically similar to the LLS in the ATM and ATM-Free treatments. The average LLS across all treatments was 7.21. The frequency of LLS in each treatment is presented in Fig. 3. No significant differences were observed among treatments (*P* = 0.159). However, 43.4% and 37.5% of the lungs in the ATM and HE treatments, respectively, had LLS ≤ 4 (low score injury), whereas 26.4% of the lungs in the ATM-Free treatment had LLS ≤ 4.

## Discussion

Currently, the use of antibiotics in livestock and the possibility of raising animals in antibiotic-free conditions have been discussed worldwide, particularly due to concerns about antimicrobial resistance and public health (Dee et al. 2018). Antibiotics are frequently used as an additional strategy to control *M. hyopneumoniae* to reduce its impact on pig performance (Maes et al. 2020). In our study, pigs receiving herbal extract (HE) containing carvacrol achieved production performance comparable to those treated with in-feed antibiotics (ATM). This finding suggests that carvacrol may represent a biologically viable alternative to synthetic antibiotics for managing endemic *M. hyopneumoniae* populations during the nursery and finishing phases.

The ADG, BW, and mortality/removal rates at the end of the nursery phase in the HE treatment did not differ from those in the ATM treatment, and BW was also similar in the finishing phase. In general, the HE treatment had an intermediate effect on production performance compared to the ATM and ATM-Free treatments. However, the F/C in the HE treatment was 2.7% more efficient than in the ATM-Free treatment during the nursery phase. This aligns with previous studies demonstrating improved ADG in nursery pigs supplemented with essential oils (Li et al. 2012) and supports the evidence that plant-based additives can enhance performance. Likewise, Cheng et al. (2018) reported improved ADG in finishing pigs treated with herbal extracts or antibiotics, while other authors have observed improvements in ADG, feed efficiency, and mortality reduction when antibiotics were included in feed (Wolter and Gaines 2016; Dee et al. 2018; Faccin et al. 2020). The antibiotics potentially provide long-term protection against endemic diseases and promote better growth performance and an active immune response (Walter et al. 2000). However, there is wide variability in responses since other studies showed no effects of ATM treatment on ADG and F/G in the nursery and/or finishing phase when piglets treated with additives and/or essential oils were compared with piglets receiving ATM in their feed (Gavioli et al. 2013; Tutida et al. 2021; Güths et al. 2022). This variability highlights that the efficacy of antibiotic or herbal supplementation depends largely on herd health status, management practices, and pathogen challenge levels. Phytogenic additives may be particularly relevant during periods of intestinal stress or pathogen exposure, as demonstrated in an experimental *Escherichia coli* challenge model in which phytogenic supplementation mitigated microbiota disruption, preserved intestinal morphology, and reduced inflammatory response (Garavito-Duarte et al. 2025). Under the endemic conditions of the present trial, carvacrol was able to sustain pig performance at levels similar to antibiotics.

In this study, the improved performance of pigs in the ATM treatment compared to those in the ATM-Free treatment was more evident during the nursery phase. Similar results were reported by Cromwell (2002), who summarized findings of 1,194 experiments evaluating the use of antibiotics in feed. Antibiotics were more effective at improving performance in young pigs, with average daily gain and feed efficiency increasing by 16.4% and 6.9%, respectively, compared to only 4.2% and 2.2% in the finishing phase. Weaning represents a highly stressful event that affects the morphology and function of the small intestine, leading to increased diarrhea and decreased feed intake and growth (Tang et al. 2022). Antibiotics are believed to improve pig resilience by modulating gut microbiota, limiting pathogen proliferation, and influencing immune responses (Walter et al. 2000; Niewold 2007). However, antibiotic administration may also disrupt beneficial microbiota, potentially leading to microbial dysbiosis and increasing susceptibility to secondary infections (Correa-Fiz et al. 2019; Thomason et al. 2017). Early-life dietary interventions targeting gut health are particularly relevant during the weaning period, as phytogenic compounds have been shown to improve intestinal morphology, enhance tight junction expression, and modulate microbial composition in weaned piglets (Zhao et al. 2024). Improvements in intestinal barrier function, enhanced nutrient digestibility, and reduced fecal shedding were observed in weaned piglets challenged with *Escherichia coli* and treated with phytogenic feed additives (Garavito-Duarte et al. 2025). Alterations in gut microbiota development during early life may also affect pigs’ susceptibility to *Mycoplasma hyopneumoniae* infection (Nair et al. 2019). In this context, carvacrol may offer a more balanced approach by supporting intestinal health and maintaining performance while potentially limiting the negative impacts on microbiota diversity commonly associated with antibiotic use (Lambert et al. 2001; Costa et al. 2013; Omonijo et al. 2018). This mechanism is consistent with studies showing that carvacrol reduces oxidative stress and inflammatory cytokine expression, thereby contributing to improved gut and lung health (Feng and Jia 2014; Carvalho et al. 2019, 2020).

It is important to highlight that the ADG improved when pigs were treated with ATM, i.e. greater ADG occurred in the nursery from 49 to 63 doa and in the finishing phase from 98 to 107 doa, which coincided with the time when the animals were given feed supplemented with ATM. This effect could not be observed in the HE treatment since carvacrol was continuously administered from weaning to slaughter. Thus, based on our performance results, we hypothesize that the absence of differences between the ATM and HE treatments could, at least in part, be explained by modulation of the gut microbiota, reducing pathogenic challenge and enhancing the anti-inflammatory properties of the carvacrol (Jamroz et al. 2006; Niewold 2007). These findings are supported by experimental evidence indicating that phytogenic compounds can modulate inflammatory signaling pathways and promote a more stable gut microbial ecosystem, thereby contributing to improved nutrient utilization and immune competence (Zhao et al. 2024). These effects may improve the availability of essential nutrients for absorption by the digestive system, improving growth performance and helping the immune response (Windisch et al. 2007; Costa et al. 2013). As recently reviewed by Nhara et al. 2025, phytogenic feed additives have been reported to enhance nutrient digestibility and improve feed conversion efficiency through modulation of gastrointestinal secretions and microbial fermentation patterns. Such mechanisms provide biological plausibility for the comparable growth performance observed between HE and ATM treatments in the present study. Thus, corroborating previous studies, our trial showed that the use of carvacrol could be considered as a substitute for in-feed antibiotics without significant impacts on performance. The only negative effect of the HE treatment was a higher cumulative percentage of mortality and removal compared to the ATM treatment. In this case, the removal of weak pigs was the main contributor to this variable. It is possible that more vulnerable individuals within the population responded differently to dietary supplementation, which may partially explain the higher removal rate observed in the HE treatment.

The PCR results and macroscopic lung lesions at slaughter suggested that neither ATM nor carvacrol in the feed prevented *M. hyopneumoniae* infection. However, the use of ATM in-feed resulted in a lower number of pigs positive for *M. hyopneumoniae* in the finishing phase compared to no ATM. This suggests that antibiotics may reduce bacterial load and delay lesion development, rather than fully preventing infection (del Pozo Sacristán 2014). On the other hand, lung lesion score and the prevalence of lungs infected with *M. hyopneumoniae* were similar regardless of the use of ATM or carvacrol, which may be due to the development of new lesions after the withdrawal of ATM beyond 162 doa (Burch 2012). However, it is important to note that lung lesion score tended to be better in the ATM treatment compared to the ATM-Free treatment. The ATM treatment produced results similar to those of the HE treatment, and the prevalence of lungs with low lesion scores was higher in the ATM and HE treatments, suggesting that the use of carvacrol may control *M. hyopneumoniae* infection similar to ATM.

In summary, we suggest that there is an opportunity to use carvacrol during the nursery phase. In the finishing phase, the removal of ATM did not adversely influence pig growth and health, but the impact on the percentage of pigs with low performance (removals) should be considered. In our study, these pigs were removed from the trial, but they were still sold by the slaughterhouse. Therefore, they should be considered in the determination of the benefits of an antibiotic-free production system. In conclusion, removing ATM from the feed for pigs in herds with endemic *M. hyopneumoniae* reduces the pig performance during the nursery and finishing phases and tends to result in larger lung areas suggestive of pneumonia at slaughter. On the other hand, our study showed that the herbal extract carvacrol can be considered an alternative to in-feed ATM without significant impacts on performance, except for the weakling removal rate, which was higher in pigs treated with carvacrol compared to ATM in the finishing phase.

## Data Availability

The data that support the findings of this study are available from the corresponding author upon plausible request.
